# Differences in Marital Satisfaction and Intention for Subsequent Childbirth by Latent Profile of Family Values among Parents with Young Children in Korea

**DOI:** 10.3390/bs14100868

**Published:** 2024-09-25

**Authors:** Youseok Choi, Ji-young Lim

**Affiliations:** Department of Child and Family Studies, Kyungpook National University, Daegu 41566, Republic of Korea; seok12022@naver.com

**Keywords:** family values, marital satisfaction, intention for subsequent childbirth, low birth rate, latent profile analysis

## Abstract

Background: The focus is on family values, marital satisfaction, and the intention for subsequent childbirth. By classifying parents based on family values and examining marital satisfaction and the intention for subsequent childbirth within these groups, the study will provide insights into future childbirth trends and identify factors influencing the intention for subsequent childbirth among different groups. Methods: This study was conducted with parents of children aged 0 to 5 attending six daycare centers in Korea, using a questionnaire to gather responses regarding family values, intention for subsequent childbirth, and marital satisfaction. Descriptive statistics were employed to analyze the characteristics of the key variables, and latent profile analysis was conducted to classify latent groups based on family values. In the verification of differences, the three-step approach was used. Results: First, the latent profile analysis revealed three groups: the ‘neutral values group’ with lower emphasis on gender roles compared to other group, the ‘individualistic values group’ with lower values on marriage and children but higher emphasis on gender roles, and the ‘family-centered values group’ characterized by higher levels of various sub-factors in overall family values. Second, comparing marital satisfaction and intention for subsequent childbirth among latent groups, the FCVG showed significantly higher levels than the TVG and the IVG. Conclusions: In their marriages, couples differed in the values they held regarding parenting, marriage, and children. Therefore, maintaining strong values in marriage and parenting and establishing a family serve as the foundation for realizing new selfhood through parenthood. Simultaneously, forming values within the new roles of being a spouse and a parent is crucial.

## 1. Introduction

South Korea’s rapidly declining fertility rate is one of the major issues facing the country, leading to a declining population, an ageing population, and limited social activities within the family unit. The decrease in births can cause various social problems, such as a reduction in the labor force and economic growth slowdown, or the limitation of family-based social activities, thereby creating social gaps [1]. The government began addressing population issues like low birth rates and aging in the early 2000s. In 2005, it established the first Basic Plan for Low Fertility and Aging Society and implemented various policies with significant budgets. For instance, the budget for the First Plan for Ageing Society and Population (2006–2010) was KRW 8.93 trillion (about USD 6.43 billion), which increased to approximately KRW 51.7 trillion (about USD 37.4 billion) annually by 2022 [2].

To conduct research aimed at solving the low birth rate issue, it is essential first to review the existing measures. The first Basic Plan for Low Fertility and Aging Society (2006–2010) focused on creating a social atmosphere to respond to these issues. The Second Plan for Ageing Society and Population (2011–2015) emphasized strengthening legal, institutional, and financial foundations, while the Third Plan for Ageing Society and Population (2016–2020) included policies addressing social structural factors such as employment, housing, and education, with initiatives like youth job creation and customized housing support for newlyweds [3]. The Fourth Plan for Ageing Society and Population (2021–2025) aims to improve the quality of life based on the life cycle of individuals, reducing childcare burdens to create a sustainable society [4].

Accordingly, measures encouraging various forms of flexible work arrangements to balance work and daily life have been established, making decisions about childbirth a comprehensive consideration of past history, current evaluations, future outlooks, and cultural and social environments [5]. In the past, it was believed that the quality of life for couples could be maintained by raising children, promoting personal growth, maturity, and adaptation. However, today’s human developmental perspective shows that becoming a parent is not the only way to experience growth, maturity, and well-being, considering various personal, historical, and social constraints and flexibility [6]. Therefore, this study aims to examine the family values of young Korean parents in the face of the declining birthrate, and then discuss the strategies for responding to the declining birthrate from a multifaceted perspective, thereby providing a basis for new academic discourse in the field of marriage and family in future research.

This shift indicates an increase in couples deciding whether to have children from a perspective of choice, unlike the past when childbirth was seen as a natural progression post-marriage. The birth statistics of 2021 [7] show that the proportion of first children has steadily increased from 2011 to 2021, while the proportion of second and third children has steadily decreased since 2014. This indicates an increase in families raising only one child after marriage compared to the past. However, the actual number of children desired by married families is different from the statistical increase in single-child households. The 2021 Population Trends Survey shows that the ideal number of children was 2.16 as of 2019, suggesting that while there is an increase in households with no children or only one child, there is still a high possibility of future childbirth plans.

Therefore, this study focuses on parents who already have children and aims to analyze their family values and contextualize their intention to have subsequent children and marital satisfaction. Becoming a parent from a couple shapes the beliefs and perceptions of how children should be developed and raised, and these beliefs can change over time based on personal experiences and values [8]. Couples may experience differences in child care before and after having their first child, creating a harmonious family atmosphere through shared parenting [9], differing from childless couples.

Research by Kim and Kim [10] indicates that more factors are considered when deciding to have the first child compared to subsequent children. Socioeconomic variables related to childbirth suggest that parents with young children have likely experienced changes in their values and satisfaction with marriage and children through the process of having children.

Thus, policies addressing childbirth should consider parents with more than one child from a different perspective than childless couples, recognizing that factors influencing childbirth decisions vary by the number of children. Understanding these differences can lead to more effective solutions to the low birth rate issue. According to National Statistical Office (2022) [11], significant changes in attitudes towards marriage and childbirth, the pursuit of new lifestyles, and the spread of gender equality consciousness are identified as major causes of the sharp decline in birth rates since the mid-1980s. In an era where low birth rates are a national issue, flexible attitudes towards marriage and increased equity in the division of roles within the family are emerging as new patterns of married life. This study aims to understand family values, including these factors, to classify groups and examine differences.

As mentioned earlier, childbirth is an elective value rather than an essential element of life. Changes in parental values are crucial in approaching and fundamentally solving the low birth rate problem [12]. Understanding the comprehensive attitudes or perspectives on life aspects such as marriage, gender roles, childbirth, and child-rearing held by parents with young children is necessary. Specifically, family values, referring to people’s perspectives or attitudes toward forming and maintaining families, serve as criteria for judging what is desirable and appropriate regarding family-related issues, often categorized into sub-concepts like views on marriage, divorce, and children [13].

Family values of parents with young children are important variables connected to better mental health and quality of life, reshaping family concepts and enhancing family health, significantly influencing future family formation. This study will examine family values divided into views on marriage, children, and gender roles. These three factors are influenced by personal beliefs and values and the pervasive societal value norms. Views on marriage include the necessity of marriage, spouse selection, cohabitation, and family planning post-marriage [14], and views on children, encompassing psychological and economic value and satisfaction, can reduce the intention to have children [15]. The equity in the division of roles between men and women in the family is an increasingly important variable influencing married life [16]. Therefore, the family values components used in this study were set accordingly.

In the socio-cultural history of Korea, influenced by Confucian culture and patriarchal systems, gender equality, role-sharing between spouses, and the pursuit of individual values will affect the changing family values through constant interaction between individuals and society [17]. Different groups can form as a result. The acceptance of changes in family values can also vary according to experiences and positions, even among parents of the same generation. Therefore, to solve the low birth rate problem, it is necessary to understand the family values of couples in more detail and examine the differences in variables related to low birth rates according to their latent group characteristics.

One of the essential factors to consider after classifying groups based on family values in addressing the low birth rate issue is the intention of parents for subsequent births. The intention to have children is not just a simple psychological factor but also a desire and will to have children within the socioeconomic and policy context, which can lead to socio-structural changes. The desire and will to have children precede the actual occurrence of childbirth [18]. Positive images of childbirth and reduced anxiety about pregnancy and childbirth can result from expressing one’s intention or attitude toward having children. However, emotional anxiety and weakened values can negatively impact the willingness for subsequent births among married women due to uncertainty about the future and reduced quality of life. Therefore, understanding attitudes toward marriage and children and preparing educational and intervention measures for positive change and objectively analyzing the intention to have children are necessary. Examining the classification of parents with young children based on family values and their subsequent childbirth intentions can help predict future births and identify factors that can encourage subsequent births among parents in each group.

Next, this research examines marital satisfaction, a crucial factor in the lives of young couples. Marital satisfaction represents an overall evaluation of married life, including the alignment between the reality and expectations of marriage. It is often categorized into marital success, marital stability, marital happiness, and marital adjustment [19]. Marital satisfaction is determined by how well individuals meet their expectations regarding marriage and family life and is influenced by factors such as communication, conflict resolution behaviors, personality, and values, and psychological independence from the family of origin [20]. Particularly, marital satisfaction varies according to individualistic, open, stable, and conservative values [21], suggesting that marital satisfaction can differ based on the family values individuals hold. The gradual decline in marital satisfaction in the years following the birth of a child suggests that marital satisfaction among Korean couples typically declines during the childbearing and parenting years [22], so it is important to explore how family values affect marital satisfaction.

The purpose of this study is to understand the changing family values of parents with young children to determine their intentions for subsequent births and to foster family health through policy support. By examining the influence of family values on intentions for subsequent births, we aim to strengthen family bonds, enhance marital satisfaction, and address the low birth rate problem. This study will analyze how marital satisfaction among parents with young children may vary according to different family values and provide insights into improving marriage satisfaction and promoting higher birth rates.

## 2. Methods

### 2.1. Participants and Design

Data for this study were collected through a questionnaire method, in which parents responded to items about family values, intention for subsequent childbirth, and marital satisfaction. The survey was administered to parents of young children attending daycare centers in Korea, after obtaining ethical approval from the institutions. The study included 324 parents from 6 daycare centers.

The average age of the fathers was 38.1 years, with the majority in their 30s. The average age of the mothers was slightly lower at 35.8 years, also predominantly in their 30s. The highest educational attainment for fathers was university graduation (79.7%), followed by high school graduation (17.4%) and postgraduate education (2.9%). Similarly, for mothers, the highest educational attainment was university graduation (74.0%), followed by high school graduation (18.1%), postgraduate education (6.0%), and middle school graduation (2.0%). Regarding the number of children, most parents had two children (161 parents, 49.6%), followed by those with one child (138 parents, 42.2%) and more than three children (27 parents, 8.2%).

### 2.2. Measures

#### 2.2.1. Family Values

In this study, the scale used in Park’s [23] research was modified and supplemented to measure the family values of parents with young children. This scale comprises a total of 28 items, covering aspects such as gender equality awareness, gender role flexibility, the necessity of marriage, the value of marriage, the necessity of children, and the value of children. Each item is rated on a 5-point Likert scale, ranging from ‘strongly disagree’ (1 point) to ‘strongly agree’ (5 points), with higher scores indicating stronger adherence to each value. Example items include statements like ‘marriage is a must,’ and ‘people with children feel less lonely in old age.’ The reliability of the scale, as indicated by Cronbach’s α, is 0.71.

#### 2.2.2. Marital Satisfaction

To measure the marital satisfaction of parents with young children, a questionnaire originally developed by Roach et al. [24] was employed. This scale comprises two sub-variables: 14 items on overall marital satisfaction and 5 items on socio-psychological homogeneity, totaling 19 items. Each item is rated on a 5-point Likert scale, ranging from ‘strongly disagree’ (1 point) to ‘strongly agree’ (5 points), with higher scores indicating higher marital satisfaction. Example items include ‘my marriage becomes more satisfying as time goes by’ and ‘my spouse encourages me to do my best.’ The reliability of the scale, indicated by Cronbach’s α, is 0.94.

#### 2.2.3. Intention for Subsequent Childbirth

To measure the intention for subsequent childbirth based on current conditions for this study, the scale developed by Kwon [25] was refined and employed. This scale comprises three sub-factors: intention for childbirth with reduced childcare stress, intention for childbirth with improved childcare facilities, and intention for childbirth with enhanced childcare policies. Each item is rated on a 5-point Likert scale, ranging from ‘strongly disagree’ (1 point) to ‘strongly agree’ (5 points), with higher scores indicating a higher intention for subsequent childbirth. Example items include ‘I would consider having more children if childcare were not physically and mentally exhausting’ and ‘I would consider having more children if parental leave policies were diversified and work arrangements were flexible.’ The reliability of the scale, indicated by Cronbach’s α, is 0.95.

#### 2.2.4. Data Analyses

First, frequency analysis was conducted to determine the general characteristics of the study subjects. Second, descriptive statistics analysis was performed to identify the characteristics of the key variables. Third, latent profile analysis (LPA) was used to classify latent groups based on the family values of parents with young children. To determine the optimal number of latent groups, the study incrementally increased the number of groups from 2 to 6, selecting the best model by considering various indices. The model’s fit was assessed using information criteria such as AIC (Akaike information criterion), BIC (Bayesian information criterion), and SABIC (sample-size adjusted BIC), looking for decreasing values to confirm model fit. Additionally, the study used the entropy index, LMR-LRT (Lo–Mendell–Rubin adjusted likelihood ratio test), BLRT (parametric bootstrap likelihood ratio test), and the smallest group’s case numbers. An entropy index close to 1 indicated high-quality latent group classification. For model comparison, indices compared models with n and n − 1 groups, selecting the model with n groups if the *p*-value was significant. The smallest group was considered appropriate if it had a classification rate of over 1% or a sample size of 25 or more [26]. For difference verification, the 3-step approach was used to control the impact of difference verification variables on latent class classification [27]. The 3-step approach has the advantage of ensuring that neither predictor nor outcome variables influence the classification of a latent profile, as the latent profile predictive and outcome variable and influence validation are performed independently.

## 3. Results

### 3.1. Descriptive Statistical Analysis

The descriptive statistics of the key variables used in this study are shown in Table 1. To confirm the normality of the key variables, skewness and kurtosis values were examined. The results showed that the absolute values of skewness and kurtosis were less than 2 and 7, respectively, indicating that all variables met the assumption of normal distribution [28].

Table 2 shows the results of the latent profile analysis based on family values. Examining the changes in information criteria, it was found that the decrease was most significant when the number of latent classes increased from two to three, and the rate of decrease slowed down, becoming more gradual after three latent classes. As the number of latent classes increased, the AIC, BIC, and SABIC values, which are information criteria, decreased. The LMR-LRT value was statistically significant at the 0.05 level in the model with three latent classes but not significant in models with more than three latent classes. The BLRT value was significant for all models. When examining the number of cases in the smallest class, models with four or more latent classes had less than 25 cases each, which was deemed inappropriate. The model with three latent classes had 43 cases (13.271%), meeting the criteria for the minimum number of cases. Therefore, considering the information criteria, classification quality, model comparison indices, and the number of cases in the smallest class, the model with three latent classes was deemed the most appropriate for the family values of parents with young children.

### 3.2. Characteristics of Each Latent Profile

**Figure 1:**
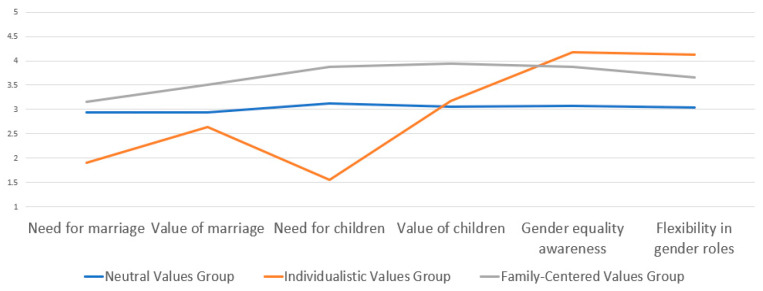
Means of indicators by family values profiles.

The characteristics of the marriage values of parents with young children, categorized into three latent profiles, are shown in Table 3 and Figure 1. Profile 1, consisting of 122 individuals (37.654%), showed intermediate levels of the necessity of marriage, the value of marriage, and the necessity of children compared to Profiles 2 and 3, with the lowest levels of the value of children, gender equality consciousness, and flexibility in gender roles. This profile was labeled the ‘neutral values group’. Profile 2, comprising 159 individuals (49.074%), had the lowest levels of the necessity of marriage, the value of marriage, and the necessity of children, but the highest levels of gender equality consciousness and flexibility in gender roles, earning the label ‘individualistic values group’. Profile 3, consisting of 43 individuals (13.271%), had the highest levels of the necessity of marriage, the value of marriage, the necessity of children, and the value of children, and was labeled the ‘family-centered values group’.

### 3.3. Differences in Marital Satisfaction and Intentions for Subsequent Childbirth among Latent Classes Based on Family Values

The construct reliability (CR) of marital satisfaction was 0.971 and the average variance extracted (AVE) was 0.946, while the CR of intention for subsequent childbirth was 0.813 and the AVE was 0.928. Conceptual reliability was above 0.70 and AVE was above 0.50, indicating that there was no problem with the convergent validity. The correlation coefficient between marital satisfaction and intention for subsequent childbirth was 0.128 and the square of this value was 0.016. The AVE value of marital satisfaction was 0.971 and the AVE value of intention for subsequent childbirth was 0.946, and both AVE values were greater than the square of the correlation coefficient, so discriminant validity was determined.

Table 4 shows the differences in marital satisfaction and intentions for subsequent childbirth among the latent classes based on family values. Examining the differences in marital satisfaction among the family values latent classes, the ‘family-centered values group’ (M = 4.279, SE = 0.101) had the highest marital satisfaction, followed by the ‘individualistic values group’ (M = 3.401, SE = 0.073) and the ‘neutral values group’ (M = 3.253, SE = 0.077). Pairwise comparisons between the latent classes showed that the differences between the ‘neutral values group’ and the ‘family-centered values group’ as well as between the ‘individualistic values group’ and the ‘family-centered values group’ were significant, but the difference between the ‘neutral values group’ and the ‘individualistic values group’ was not significant. Thus, the marital satisfaction of the ‘family-centered values group’ was significantly higher than that of the ‘neutral values group’ and the ‘individualistic values group’.

Examining the differences in intentions for subsequent childbirth among the family values latent classes, the ‘family-centered values group’ (M = 3.635, SE = 0.208) had the highest intentions, followed by the ‘individualistic values group’ (M = 2.935, SE = 0.111) and the ‘neutral values group’ (M = 2.893, SE = 0.098). Pairwise comparisons between the latent classes showed that the differences between the ‘neutral values group’ and the ‘family-centered values group’, as well as between the ‘individualistic values group’ and the ‘family-centered values group’’, were significant, but the difference between the ‘neutral values group’ and the ‘individualistic values group’ was not significant. Thus, the intentions for subsequent childbirth of the ‘family-centered values group’ were significantly higher than those of the ‘neutral values group’ and the ‘individualistic values group’.

## 4. Discussion

In this study, the latent groups were classified based on the family values of parents with young children aged 0–5 in Korea. Differences in marital satisfaction and intentions for subsequent childbirth according to the classified profiles were also examined. The results are discussed as follows.

First, a latent profile analysis was conducted based on the necessity and value of marriage, the necessity and value of children, awareness of gender equality, and flexibility in gender roles. This analysis revealed three latent groups, which were named the ‘neutral values group’, the ‘individualistic values group’ and the ‘family-centered values group’. The ‘neutral values group’ showed moderate levels of necessity and value of marriage and children, but the lowest levels of gender equality awareness and gender role flexibility compared to the other groups. The ‘individualistic values group’ comprising 159 individuals (49.07%), had the highest levels of gender equality awareness and gender role flexibility but the lowest levels of necessity and value of marriage and children. The ‘family-centered values group’ exhibited the highest levels in all areas except for gender equality awareness and gender role flexibility.

The finding that there are three distinct latent groups with different family values among parents of young children suggests that even parents with similarly aged children can have varying family values. The ‘neutral values group’ prioritizes stability in marriage, forming new families, and independence through marriage but differentiates between the roles of husbands and wives in domestic and livelihood responsibilities. In Korea, traditional family values are sometimes viewed as emphasizing the importance of family over the individual, requiring continuous effort from family members to maintain cohesion [29]. The study found that the ‘neutral values group’ exhibited relatively traditional Korean value characteristics, even if they were neutral with respect to a sense of family community through marriage and children and consideration of gender equality or gender role flexibility in the home.

However, the ‘individualistic values group’ can be interpreted as prioritizing self-realization over marriage and children, breaking away from the gender role stereotypes influenced by Korea’s patriarchal culture and the prescribed behaviors within the current cultural context. Ogihara [30] found that individuals in Asian countries develop individualistic values as they struggle to adapt to rapidly changing environments. The decline in multigenerational households, the rise in single-person households, and increasing divorce rates are seen as patterns of growing individualism. Korea exhibits similar social trends, where individualistic skills such as autonomy, self-expression, and self-assertion are considered more suitable for survival and success in new, opportunity-filled, competitive urban environments [31]. Therefore, this study’s results indicate that many parents have formed individualistic values, moving away from patriarchal elements and adopting a more individualistic approach.

Secondly, examining marital satisfaction and intentions for subsequent childbirth across the latent family value groups revealed that the ‘family-centered values group’ had significantly higher levels compared to the other two groups. In fulfilling the roles of spouse and caregiver within a household the ‘neutral values group’ identified in this study prioritizes family over the individual, viewing life through a family-centered lens. They define roles in childcare domestic work in a traditional manner, which can lead to marital dissatisfaction and stress from caregiving responsibilities, resulting in lower marital satisfaction. However, while the ‘neutral values group’ in this study scores lower in certain areas, it shows signs of adopting a more gender-equal culture compared to past patriarchal norms. Thus, this group appears to be gradually embracing a more egalitarian approach within the household, indicating potential for positive outcomes in terms of marital satisfaction and childbirth intentions if gender-equal practices continue to be adopted.

Furthermore, whether men and women perceive gender equality within their marriage and view themselves as individual entities separate from their roles as spouses and parents influences the development of individualistic family values [32]. So, in cases where roles within marriage are seen as inflexible and unequal, or if personal value and self-fulfillment are not achieved, marital satisfaction tends to be lower. In addition, strong individualism can lead to feelings of unhappiness due to a lack of social support, increased competition, and excessive comparison with others [33]. This, coupled with the prevalent use of social media platforms like Instagram and YouTube, where comparisons to others’ lives are common, can amplify dissatisfaction with one’s own marriage. In essence, how individuals balance personal life and self-fulfillment within the roles of spouse and parent affects marital satisfaction, indicating that sacrifices, comparisons, and the absence of gender equality in marriage should be minimized, while achieving personal growth and fulfillment through children should be emphasized.

The intention for subsequent childbirth, defined as the desire or consideration for having more children among married women with one child, is influenced by economic conditions, parenting values, self-fulfillment needs, and personal experiences [34]. Both the ‘neutral values group’ and the ‘individualistic values group’ have significantly lower perceptions of the necessity of children compared to the ‘family-centered values group’, which can contribute to their reluctance to have more children. This suggests the need for strategies to enhance the perceived role of children and improve parental quality of life through better support and management.

On the other hand, there was no significant difference in marital satisfaction and subsequent childbearing between the ‘neutral values group’ and the ‘individualistic values group’. This indicates that, given the high cost of marriage, childbirth, and childrearing in Korean society, phenomena such as marital conflict and negative thoughts about and reluctance to have a second child cannot be viewed as simply the result of differences in values, as parents are paying a significant amount of money for the process of marriage and childrearing, starting with wedding financing.

The limitations and recommendations of this study are as follows: first, this study only included marriage, children, and gender roles in family values, but there may be other factors that make up family values, so future studies should explore the sub-factors that shape family values. Second, this study examined family values among parents of infants and toddlers, but family values may vary by family members and time period, such as among those in early adulthood before marriage and childless couples. In addition, marital satisfaction and subsequent fertility may also vary by parental demographic differences, such as parental gender, age, infancy, and toddlerhood. Future research should examine how each of these variables changes over time among parents of young children, and further research is needed on the various variables that may influence the three variables, as well as appropriate control variables.

In the midst of rapid social change, family values are expanding and changing, and various factors such as intergenerational conflict and gender conflict are affecting young people’s attitudes toward marriage and childbearing and their quality of life. Therefore, Korea’s population policies and approaches to marriage and childbearing should adopt a new paradigm based on family values to understand the diverse values of young people, and the findings of this study on marital satisfaction and subsequent childbearing according to family values can provide a useful basis for developing policies to resolve conflict points caused by differences in couples’ family values.

## 5. Conclusions

The intention for subsequent childbirth is influenced by various factors, such as economic conditions, the perceived value of children, parenting philosophy, self-realization needs, and personal experiences. Both the ‘neutral’ and ‘individualistic’ groups exhibited significantly lower perceptions of the necessity of children compared to the ‘family-centered’ group, contributing to their reluctance to have more children. This indicates the need for strategies to enhance the perceived role and support of children, thereby improving parents’ quality of life and increasing their willingness to have more children.

These results underscore the importance of considering changing family values when formulating policies related to marriage and childbirth. While high values for marriage and children persist, the sense of obligation to marry or have children is weakening. This highlights the need for policies that address the utility and value of marriage and children in individuals’ lives. Particularly, fair distribution of household chores and the implementation of gender-equal policies can influence future childbirth plans. However, the findings also indicate that high levels of gender equality alone do not necessarily lead to positive outcomes. Excessive division of roles and the pressure of balancing work and family life can contribute to negative perceptions and mistrust about the feasibility of maintaining complementary roles within the family. Therefore, mutual support, understanding, and trust between partners are essential for improving marital satisfaction and childbirth intentions.

Currently, the average age at marriage continues to rise, and despite being married, individuals may not directly perceive the value of maintaining their marriage or raising children. The increasing age at childbirth and declining satisfaction with marriage and the value of children can negatively impact future childbirth plans. Therefore, low birth rate policies should address both unmarried and married individuals, focusing on fulfilling their self-realization and the value of children after marriage.

The results of this study are significant in that they examine the family values of parents with young children in a low birthrate and aging society by categorizing them into distinct groups. This approach reveals the characteristics and differences of each group and offers a new perspective on the factors influencing subsequent childbirth. Additionally, it highlights the importance of forming values within the family by finding satisfaction and stability in family life and realizing new aspects of self-fulfillment within the roles of spouse and parent. The study shows that marital satisfaction and the intention for subsequent childbirth vary according to the family values of parents with young children, indicating that these factors can differ based on the role values attributed to men and women, fathers and mothers, and husbands and wives in marriage and child-rearing.

## Figures and Tables

**Table 1:** Variables descriptive statistics number of latent classes according to family values.

	M	SD	Skewness	Kurtosis
Need for marriage	2.47	0.87	−0.136	−0.442
Value of marriage	2.87	0.52	−0.015	0.854
Need for children	2.46	1.23	0.358	−0.925
Value of children	3.23	0.47	0.478	0.443
Gender equality awareness	3.72	0.84	−0.123	−0.529
Flexibility in gender roles	3.65	0.81	−0.208	−0.357
Marital satisfaction	3.46	0.84	−0.220	0.455
Intention for subsequent childbirth	3.01	1.20	−0.093	−0.798

Note: *N* = 324.

**Table 2:** Model fit indices for latent profile analyses.

		Number of Groups					
		Class 1	Class 2	Class 3	Class 4	Class 5	Class 6
AIC		4441.965	4119.884	4000.335	3935.25	3885.092	3838.625
BIC		4487.334	4191.718	4098.635	4060.015	4036.321	4016.32
SABIC		4449.271	4131.452	4016.165	3955.342	3909.445	3867.24
Entropy		-	0.783	0.827	0.847	0.875	0.891
LMR-LRT		-	0.001	0.016	0.087	0.089	0.003
BLRT		-	0.000	0.000	0.000	0.000	0.000
Class Proportion (*N*)	1	100 (324)	48.456 (157)	37.654 (122)	6.481 (21)	6.172 (20)	0.617 (2)
	2		51.543 (167)	49.074 (159)	35.493 (115)	36.419 (118)	36.419 (118)
	3			13.271 (43)	14.506 (47)	6.790 (22)	6.790 (22)
	4				43.518 (141)	8.950 (29)	42.901 (139)
	5					41.666 (135)	6.481 (21)
	6						6.790 (22)

Note: *N* = 324.

**Table 3:** Average scores and composition ratios of family values by latent profile.

	M(SE)						*N* (%)
	1	2	3	4	5	6	
Profile 1	2.945 (0.070)	2.947 (0.031)	3.116 (0.137)	3.052 (0.041)	3.081 (0.084)	3.037 (0.081)	122 (37.654)
Profile 2	1.908 (0.080)	2.638 (0.049)	1.559 (0.088)	3.169 (0.037)	4.480 (0.061)	4.131 (0.056)	159 (49.074)
Profile 3	3.156 (0.164)	3.516 (0.105)	3.867 (0.240)	3.942 (0.084)	3.882 (0.195)	3.657 (0.213)	43 (13.271)

Note: *N* = 324. 1 = Need for marriage. 2 = Value of marriage, 3 = Need for children, 4 = Value of children, 5 = Gender equality awareness, 6 = Flexibility in gender roles

**Table 4:** Differences in marital satisfaction and intention for subsequent childbirth between groups.

	M (SE)			Pairwise Comparison between Latent Groups	
	Group 1 (n = 122)	Group 2 (n = 159)	Group 3 (n = 43)	Comparison	**χ** ^2^
Marital satisfaction	3.253 (0.077)	3.401 (0.073)	4.279 (0.101)	Overall test	67.794 ***
				Profile 1 vs. Profile 2	1.753
				Profile 1 vs. Profile 3	60.624 ***
				Profile 2 vs. Profile 3	48.556 ***
Intention for Subsequent Childbirth	2.893 (0.098)	2.935 (0.111)	3.635 (0.208)	Overall test	10.232 **
				Profile 1 vs. Profile 2	0.074
				Profile 1 vs. Profile 3	9.624 ***
				Profile 2 vs. Profile 3	8.531 ***

** *p* < 0.01, *** *p* < 0.001.

## Data Availability

The dataset for this study is available from the authors immediately upon request.
